# Allergic reactions against Covıd-19 vaccines

**DOI:** 10.3906/sag-2104-329

**Published:** 2021-10-21

**Authors:** Hilal ÜNSAL, Bülent Enis ŞEKEREL, Ümit Murat ŞAHİNER

**Affiliations:** 1 Department of Pediatric Allergy, Faculty of Medicine, Hacettepe University, Ankara Turkey

**Keywords:** COVID-19, vaccine, allergy, hypersensitiviy, polyethylene glycol

## Abstract

Coronavirus Disease 2019 (COVID-19) affected the whole world in a short time. One of the most influential public health initiatives modern medicine has to offer, the vaccine has become even more important as the COVID-19 pandemic continues to worsen worldwide. Many vaccine trials were launched during the COVID-19 pandemic, and these vaccines were widely used around the world, offering realistic hope for ending the pandemic. Allergic reactions to vaccines were reported shortly after their approval. These reactions, in general, are rare, but, in some circumstances, they can be serious. Allergy to vaccines can occur because of either the active vaccine component or vaccine ingredients. The spectrum of the reactions may be just a local hypersensitiviy reaction or may be as severe as an anaphylaxis, which is an acute severe, life-threatening systemic hypersensitive reaction, and it requires quick intervention. If an allergy is suspected, a correct examination followed by algorithms is important for true diagnosis, treatment, and decision regarding revaccination. Patients who experience an allergic reaction with the first dose of covid 19 vaccine should be directed to the allergy-immunologist, and the evaluation of at-risk patients should be individualized. Finally, we should point out that the benefits of current COVID-19 vaccines go far beyond the side effects, and that the vaccine is the most important way to recover from the pandemic.

## 1. Introduction

Routine immunization has effectively reduced death and morbidity rates because of a variety of infectious diseases, and it is one of the most effective public health interventions [1,2]. Severe allergic reactions to vaccines are rare and difficult to predict. In general, anaphylactic reactions to vaccinations occur at a rate of about 1 per million.[3, 4] Vaccination has become increasingly important as the global pandemic, the 2019 coronavirus disease (COVID-19), continues to worsen worldwide. After the COVID-19 pandemic, many vaccine studies began worldwide, and vaccines such as Pfizer/BioNTech BNT162B2, Moderna mRNA-1273 and AstraZeneca recombinant ChAdOx1-S were first approved. Although rare, severe allergic reactions to mRNA-based vaccines were reported shortly after their approval.[4] In this review, we aimed to make an allergic reaction risk assessment related to COVID-19 vaccination and recommend a standard diagnostic and management procedure in case of reaction.

## 2. Covid-19 vaccines and allergic reactions

RNA vaccines, inactivated vaccines**, **replication-incompetent vector vaccines, and recombinant protein vaccines are different vaccine approaches produced for protection from covid 19 infection**. **BNT162b2 (Pfizer-BioNTech) and mRNA-1273 (Moderna) are mRNA vaccines, and they are delivered in a lipid nanoparticle to express a full-length spike protein. Since the approval of mRNA-based vaccines, severe general reactions have been reported from the United Kingdom and the United States. Twenty-one adverse events were detected as anaphylaxis after administration of a reported 1,893,360 first doses of the Pfizer-BioNTech COVID-19 vaccine (11.1 cases per million doses) [5, 6]. Anaphylaxis has also been reported from the Moderna mRNA COVID-19 vaccine.[7] This has triggered the need to provide recommendations on how to manage covid 19 vaccine allergic reactions.

The AstraZeneca ChAdOx1-S (Vaxzevria) vaccine, which is a recombinant chimpanzee adenoviral vector, and the Johnson & Johnson Ad26.COV2.S (Janssen) vaccine, which is a recombinant adenovirus type 26 vector, were other produced vaccines on the market. 1,2European Medicines Agency (2021). Vaxzevria COVID-19 Vaccine AstraZeneca [online]. Website https://www.ema.europa.eu/en/medicines/human/EPAR/vaxzevria-previously-covid-19-vaccine-astrazeneca. [29 January 2021].European Medicines Agency (2021). COVID-19 Vaccine Janssen(Ad26.COV2-S) [online]. Website https://www.ema.europa.eu/en/medicines/human/EPAR/covid-19-vaccine-janssen. [11 March 2021]. Approximately 6.85 million doses of Johnson & Johnson COVID-19 vaccine (Janssen) have been administered in the USA. Six cases of cerebral venous sinus thrombosis with thrombocytopenia have been reported after receiving Janssen COVID-19 vaccine. All of the cases consisted of women between the ages of 18–48, and the events occurred 6-13 days after vaccination. One patient has died. In the statement made by the FDA and the Centers for Disease Control and Prevention (CDC), it was recommended to stop the use of Janssen COVID-19 vaccine and the USA, South Africa, and the European Union decided to temporarily stop the use.^3^Food and Drug Administration (2021). Why are the FDA and CDC recommending a pause in the use of the Janssen COVID-19 Vaccine? [online]. Website https://www.fda.gov/emergency-preparedness-and-response/mcm-legal-regulatory-and-policy-framework/janssen-covid-19-vaccine-frequently-asked-questions. [14 April 2021]. 

Unusual cases of thrombotic complications along with thrombocytopenia have been observed in patients after vaccination with AstraZeneca ChAdOx1-S (Vaxzevria). The majority of these reported cases occurred within 2 weeks after vaccination and consisted of women under 60 years of age. The safety committee of EMA (Pharmacovigilance Risk Assessment Committee, PRAC) has comprehensively reviewed 62 cases of cerebral venous sinus thrombosis and 24 cases of splanchnic vein thrombosis, 18 of which were fatal. The EMA declared that, based on the currently available evidence, certain risk factors cannot be confirmed, unusual thrombotic complications with low platelets should be listed as very rare side effects of Vaxzevria,and concluded that the benefits of the vaccine continue to outweigh the risks for those receiving the vaccine.^4^European Medicines Agency (2021). AstraZeneca’s COVID-19 vaccine: EMA finds possible link to very rare cases of unusual blood clots with low blood platelets [online]. Website https://www.ema.europa.eu/en/news/astrazenecas-covid-19-vaccine-ema-finds-possible-link-very-rare-cases-unusual-blood-clots-low-blood. [07 April 2021]. The British Medicines and Healthcare Products Regulatory Agency (MHRA) stated that the benefits of vaccination for people aged 30 and over and those with other health problems outweigh the risk of coagulation problems and for people under the age of 30 who do not have other health problems, it is currently recommended that another COVID-19 vaccine be preferred over the AstraZeneca vaccine.^5^National Health Information Service (2021). Side effects of the coronavirus vaccines; Reports of very rare blood clots [online]. Website https://www.nhsinform.scot/covid-19-vaccine/the-vaccines/side-effects-of-the-coronavirus-vaccines. [14 April 2021].

## 3. Mechanisms of hypersensitivity reactions for vaccines

An allergic reaction is defined as a harmful idiosyncratic response given by an immune mechanism.[8] Allergic reactions caused by vaccines are usually of Type I and IV hypersensitivity reactions. Immunologically mediated allergic reactions are either acute in onset or delayed.[9] The majority of acute-onset reactions are type I hypersensitivity reactions.[10] Immediate reactions to vaccines is caused by the presence of IgE in a patient, which can precipitate degranulation of mast cells (MC) and release of histamine in reply to an antigen within the vaccine.[11] 

**Figure 1:**
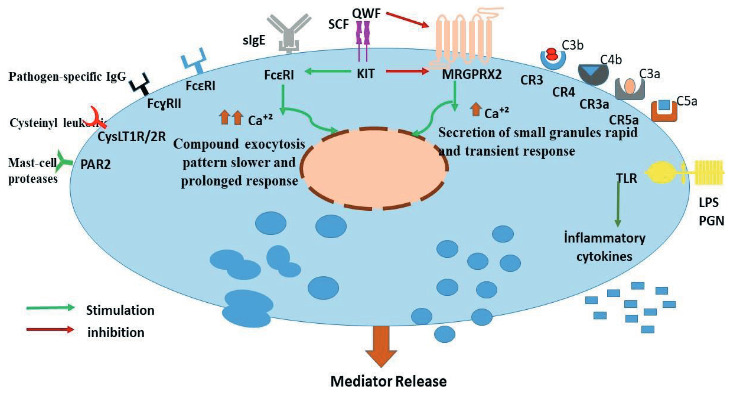
The structure of the mast cell: main receptor systems and ligands.[13]

Mast cells are thought to play a major role in the development of immediate allergic reactions, and, as upon activation, they release a kind of mediators from stored granules. Also mast cells are involved in homeostasis, inflammation, innate and adaptive immunity, angiogenesis in various tissues.[12] Therefore, they are often found at the host-environment interface, like the skin, gastrointestinal tract or lungs which are threatened by various external factors, pathogens and allergens. Main receptor systems and ligands involved in mast cell activation are shown in Figure 1.[13] Postactivation MCs release numerous mediators in immediate reaction. Histamine, proteases, heparin and prostaglandin D2, leukotriene C4, thromboxane, tumor necrosis factor alpha, etc. are among these mediators.[14]

## 4. Clinical manifestations

### 4.1. Immediate allergic reactions

Immediate hypersensitivity to vaccines is rare; however, it is important for the clinician to recognize these reactions correctly for the future vaccine administration decision. It has been stated that the average rate for immediate type reactions in children and adolescents is 0.22 per 100,000 doses of vaccinations.[15] Allergic hipersensitivite reactions to vaccines may be mild and limited in the scope of symptoms and involvement of organ systems. The most common symptoms of IgE-mediated allergic reactions are urticaria and angioedema, with less common symptoms including nasal congestion, wheezing, cough, stridor, vomiting, diarrhea abdominal pain and hypotension. Thus, typical signs of an allergic reaction occurring an onset within minutes and less than 4 h postvaccination.[16–18]

### 4.2. Anaphylaxis

Anaphylaxis is an acute severe, life-threatening generalized or systemic hypersensitive reaction*.*[19–21] Anaphylaxis is triggered by the binding of an allergen to specific immunoglubulin E (IgE). Anaphylaxis to vaccines is rare. The incidence of anaphylaxis with vaccine estimated at 1.31 in 1 million doses given.[3] Anaphylaxis is a rapidly progressive allergic reaction involving multiple organ systems such as skin, respiratory, circulatory and gastrointestinal systems. Although skin findings are the most common, skin findings may not be seen in anaphylaxis.[22–24] Symptoms of anaphylaxis generally have their onset within minutes and symptoms usually start within the first hour after immunization.[17] The faster the anaphylaxis begins, the more severe the reaction. Although uniphasic reactions are mostly seen, 20% biphasic reaction can be seen.[25] Vaso vagal reactions may occur after vaccination and can be confused with anaphylaxis.[26] Bradycardia, vasodilatation and hypotension occur and pallor, nausea, vomiting, profuse sweating, myoclonus and/or incontinence such symptoms can be seen. Hypotension may occur in both vasovagal reactions and anaphylaxis. Absence of bronchospasm and particularly cutaneous manifestations in vasovagal reactions is important in differential diagnosis. Heart rate is usually high in anaphylaxis, but bradycardia is always seen in vasovagal reactions.[25, 27] In the patient group with a history of vasovagal reaction, future vaccines should be administered while lying supine.[26]

### 4.3. Delayed reactions 

Delayed type reactions can be seen generally days after exposure, but the onset of symptoms can be as early as 6 hours or delayed for up to 2 to 3 weeks. These reactions may be nonimmunologic or immunologic. Maculopapular rash on the skin is the most common symptom of delayed-type reactions.[11, 28] Aluminum containing vaccines may cause an injection site nodule, this may be involved to delayed type hypersensitivity to aluminum.[29]

### 4.4. Local reactions

Local reactions include pain, redness, and/or swelling at injection site are common and selflimited. Large local reactions are less common and generally occur within 24–72 h after administration.[30] Mild local reactions are seen due to nonspecific inflammation both from the vaccine injection itself and from the injection of foreign substances. Therefore, the administration technique is important. Deeper injection application and injection in the thigh rather than the arm are associated with a lower incidence of local reactions.[30–32] Local reactions and constitutional symptoms, especially fever, are common after the administration of many vaccines and do not contraindicate administration of future doses of the same vaccine.[33,34]

## 5. What are the possible causes of Covid 19 vaccine reactions?

Vaccines include not only the antigen in charge of stimulating the immune response, but they also contain a number of additional ingredients. Therefore, an allergic reaction occurs not only against the active ingredient itself but also against the vaccine components. Covid 19 vaccines and their ingredients are shown in the Table 1. [35]. To date, allergic reactions mostly related to covid-19 vaccines have been attributed to two important ingredients, polyethylene glycol and polysorbate 80.

**Table 1:** Covid 19 vaccines and their ingredients [35].

Vaccine	Manufacturer	Vaccine type	Ingredients
BNT162b2	Pfizer-BioNTech	mRNA-based vaccine	§ [(4-hydroxybutyl)azanediyl) bis (hexane-6,1- diyl)bis(2-hexyldecanoate)] § 2[(PEG)-2000]-N,N ditetradecylacetamide §1,2-Distearoyl-sn-glycero-3-phosphocholine §Cholesterol §Potassium chloride, monobasic potassium phosphate, sodium chloride, dibasic sodium phosphate dehydrate §Sucrose § Water for injections
mRNA-1273	Moderna	mRNA-based vaccine	§SM-102, 1,2-dimyristoylrac-glycero-3- methoxypolyethylene glycol-2000 [PEG2000-DMG §1,2-Distearoyl-sn-glycero-3-phosphocholine §Cholesterol §Tromethamin, Tromethamin hydrocholoride, acetid acid, sodium acetate §Sucrose
ChAdOx1	AstraZeneca	Vector-based vaccine Replication-İncompetent Chimpanzee Adenovirüs vectör	§L-Histidine §L-Histidine hydrochloride monohydrate §Polysorbate 80 §Magnesium chloride hexahydrate §Sodium chloride §Disodium edetate dihydrate §Ethanol §Sucrose § Water for injections
CoronaVac	Sinovac	Inactivated SARSCoV-2 virus	§Disodium hydrogen phosphate §Sodium dihydrogen phosphate §Sodium chloride §Aluminum hydroxide

### 5.1. Polyethylene glycol (PEG)

Allergic reactions to vaccines are often due to additives, preservatives and other ingredients rather than the active ingredient itself.[10, 35] Pfizer-BioNTech and Moderna Covid-19 mRNA vaccines are not formulated with any drugs, food or latex. Both vaccines contain, in addition to the modified viral mRNA, various salts, carbohydrates and lipids and the excipient PEG (Polyethylene glycol). [36, 37] At present, there are no other vaccines that use PEG 2000. PEG, also known as macrogol, is an excipient for the purpose of stabilizing the lipid nanoparticle containing the mRNA. PEG is widely used in many daily products including; cosmetics, drugs, lozenges, laxatives, food additives and rarely trigger hypersensitiv allergic reactions.[38–44] IgE-mediated hypersensitiv reactions and anaphylaxis have been reported to PEGs of different molecular weights and the majority of reactions are due to high molecular weight PEGs.[40, 42] Otherwise, PEG can trigger non-IgE dependent complement mediated mast cell activation through IgG or IgM. This situation is called “Complement Activation-Related Pseudoallergy” (CARPA), which is caused by the activation of the complement system. These hypersensitivity reactions occur directly upon first exposure to lipid excipients, including lipid nanoparticles without prior sensitization, and the symptoms generally decrease or disappear on subsequent treatment. Thus, such immunological responses are called ‘pseudoallergy’. [37, 45, 46] 

### 5.2. Polysorbate 80

The AstraZeneca recombinant adenoviral ChAdOx1-S vaccine contain the excipient polysorbate 80. Polysorbate 80, also known as Tween 80. Polysorbate, structurally similar to PEG. Polysorbate is also an excipient in a many medical products (eg, vaccines, vitamin oils, anticancer agents), ointments, lotions, creams, and medication tablets. [36, 47] At least 70% of injectable biological agents and mAb treatments include a polysorbate. Most typically there is polysorbate 80. [48] Polysorbate 80 has been used as excipient in numerous vaccines (e.g., influenza vaccines, pneumococcal conjugate vaccine, DTaP and its analogs, HepB, HPV, zoster). [35] Polysorbate can crossreact with PEG and may cause IgE-mediated hypersensitivity reactions. [49] 

### 5.3. Patient Related risks and Covid-19 Vaccine Allergy

A history of food, venom, inhalant, latex, pet allergies and allergy to oral medications (including the oral equivalent of an injectable medication) are particularly listed as not being precautions or contraindications for receiving the covid 19 vaccine. In case of mastocytosis/mast cell activation syndromes, idiopathic anaphylaxis, severe or multiple drug allergy and previous anaphylaxis to other vaccines, patients should be referred to an allergist before vaccination, but these are not considered contraindications. These patients are considered to be observed for 30 min after vaccination, and vaccination in a hospital setting is recommended. (Table 2) [35]-6United States Centers for Disease Control and Prevention (2020) .Interim Clinical Considerations for Use of mRNA COVID-19 Vaccines Currently Authorized in the United States [online]. Website https://www.cdc.gov/vaccines/covid-19/info-by-product/clinical-considerations.html. [Reviewed December 20, 2020]. These vaccines are contraindicated in case of allergy to one of the components of the vaccine and in case of severe allergic reaction to the first dose.[35, 50]-⁶ Although no severe reaction to vaccines have been noticed, a strong relationship has been observed between anaphylaxis and asthma, particularly when asthma is not well-controlled.[51] If there is uncontrolled asthma, vaccination is recommended in a hospital setting. If patients are allergic to latex, vaccination is done only with latex-free gloves.[50] Patients with only a delayed onset local reaction around the injection site after the first vaccine dose do not have a contraindication or precaution to the future dose.⁶

**Table 2:** Allergic risk assessment for COVID-19 vaccination and suggestions during vaccination[35]-⁶.

	The Patients’ History	Suggestions
Proceed with vaccination	Food allergy Allergic asthma Allergic rhinoconjunctivits Atopic eczema Urticaria Insect venom allergy Allergy with oral medications Family history of allergiesLocal reaction history with previous vaccination	Routine vaccination with 15 minute observation [Observation time can be extended up to 30 minutes (e.g. in patients with a history of anaphylaxis)]
Vaccination with precautions	History with immediate allergic reactions(mostly anaphylaxis) to other vaccines or injectable medicationHistory of idiopathic anaphylaxis Mast cell disorders	-Referral to the allergy centre -longer observation (30 minute) and monitoring of vital signs if vaccination is to be applied
Vaccination is contraindicated	History of allergic reactions to prior dose vaccine or the vaccine ingredients	-Do not vaccine administration -Referral to the allergy centre(evaluate potential alternative Covid-19 vaccination)

## 6. Management of severe hypersensitivity reactions and anaphylaxis

Anaphylaxis is considered a medical emergency with its immediate onset and rapid progression to cardiovascular and/or respiratory collapse resulting in death within minutes of inception. Therefore, it requires quick intervention. The first thing to do is to quickly evaluate the patient’s circulation, respiration, consciousness and skin findings and to give the appropriate position. Adrenaline should be administered immediately in patients with a diagnosis of anaphylaxis or with a high probability. It should be known that delayed adrenaline therapy may cause the reaction to progress rapidly and result in death. Adrenaline administered intramuscularly (in a dose of 0.01 mg/kg of a 1:1000 [1 mg/mL] solution to a maximum of 0.5 mg in adults and 0.3 mg in children). Adrenaline should be administered intramuscularly (IM) to the middle anterolateral part of the thigh (vastus lateralis muscle).[21, 22, 52] Depending on the clinical condition of the patient, the dose can be repeated every 5–15 min. The patient should lie on her/his back and legs up. The patient’s blood pressure, heart rate, O_2_ saturation should be measured periodically, and respiratory and cardiac status should be evaluated.

**Figure 2:**
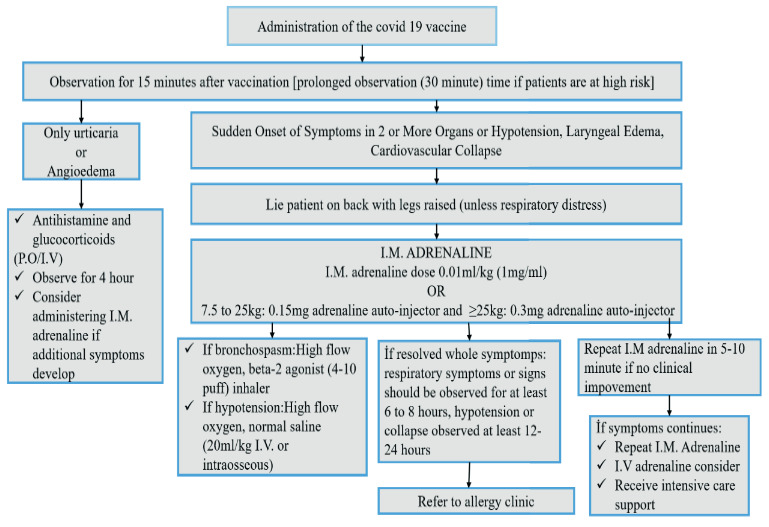
An algorythm of diagnosis and treatment for covid 19 vaccines-related reactions [19, 50].

If hypotension persists or signs of cardiovascular shock occur despite IM given adrenaline, rapid IV fluid (saline) replacement is required. Crystalloids are the fluid of choice and should be given in boluses of 20 mL/kg. [19, 53] Patients with respiratory distress should be given high flow oxygen (preferably 100% using a nonrebreather facemask).[54] In the presence of shortness of breath and wheezing despite the injection of adrenaline, inhaled beta 2 agonists can be applied.[19, 55] Antihistamines are effective on skin, nasal, and eye symptoms. However, they do not prevent the development of airway obstruction, hypotension, and shock. Therefore, they should not replace adrenaline.[56] The role of corticosteroids in the treatment of anaphylaxis is controversial. However, it has not been shown that corticosteroids reduce the severity of the reaction or prevent premature death in anaphylaxis. It should never be administered before adrenaline, and it should be known that it will not replace adrenaline in anaphylaxis.[56–58] Glucagon should be administered in cases where anaphylaxis develops while using beta blockers, if hypotension persists despite adrenaline administration. A total of 1–5 mg in adults and 20–30 mg/kg (maximum 1mg) in children should be IV administered over 5 min. Then 5–15 mg/dk IV infusion is continued.[19, 59] The algorythm of diagnosis and treatment for covid 19 vaccines related reactions is shown in Figure 2. [19, 50]

All patients with anaphylaxis should be observed until symptoms have completely resolved. Patients who presented with respiratory failure should be with care monitored for at least 6–8 hour, and patients who presented with hypotension require monitoring for at least 12–24 h.[19] Patients with a severe initial presentation or the need for more than one dose of epinephrine may be at increased risk for recurrent symptoms so an extended observation period of 24 h is recommended for patients.[50, 52] In the case of a severe anaphylactic reaction, the risk of biphasic anaphylaxis increases. Risk factors for the occurrence of biphasic anaphylaxis can be summarized as needing repeated doses of epinephrine (ie >1 dose of epinephrine), unknown trigger of anaphylaxis, large pulse pressure, drug-induced anaphylaxis in children, and cutaneous signs and symptoms.[52] On discharge, patients should be instructed to see an allergist immunologist for the future vaccination decision.

## 7. Follow up and diagnostic procedures

### 7.1.The management of patients who experience potentially allergic reactions to a COVID-19 vaccine

Patients who experience an allergic reaction with the first dose of covid 19 vaccine should be directed to the allergy-immunologist for the next dose of vaccine approach. The evaluation of at-risk patients should be individualized. Patients with a previous history of anaphylaxis against a vaccine should be revaccinated only if absolutely necessary.[30] A split-dose challenges protocol could provide an option for administration in people at higher risk for developing serious or fatal COVID-19 infections and who have already experienced a suspected or confirmed severe allergic reaction to a COVID-19 vaccine or any of its components. For other vaccines split-dose challenges (administer 0.05 mL of 1:10 dilution, and 10%, 20%, 30%, and 40% of the full dose incrementally in alternate arms at 15-min intervals, followed by a minimum 30-minute observation period) have been used.[5, 17] That it is typically performed with other vaccines, but, currently, there are no supportive efficacy or safety data for this approach.

As a different approach, patients with negative skin tests to the vaccine but with a history of anaphylaxis or severe allergic reactions can be immunized with split-dose vaccination. At first, 10% of the dose is administred, and 30 min later, the remaining 90% is applied provided that no allergic reaction has occurred following the initial dose. These vaccination approaches should only be used by experienced personnel in a controlled settings where emergency treatment of anaphylaxis is available [30].

Patients who have experienced a systemic allergic reaction to vaccine should not receive a second dose of that vaccine, nor a vaccine with similar ingredients. European Medicines Agency (EMA) and U.S. Food and Drug Administration (FDA) recommended the vaccine should not be administered if there is an allergy to one of the components of the vaccine and if there was a severe allergic reaction to the first dose. Vaccination should be administered under close medical supervision with appropriate medical attention.[50]-7European Medicines Agency (2020). EMA/660602/2020; EMEA/H/C/005735 [online]. Website https://www.ema.europa.eu/en/news/ema-recommends-first-covid-19-vaccine-authorisation-eu. [21.12.2020].For people who have a contraindication to one type of COVID-19 vaccine that is currently permitted, care should be taken to apply other vaccines. For example, patients with a contraindication to one of the mRNA Covid-19 vaccines should not receive doses of either of the mRNA vaccines. Both mRNA Covid-19 vaccines contain polyethylene glycol (PEG), and the AstraZeneca-ChAdOx1-S Covid-19 vaccine contain polysorbate 80. Cross-reactive hypersensitivity may occur between PEG and polysorbate, but after appropriate precautions have been taken, in case of a contraindication to mRNA COVID-19 vaccines, people may be able to receive Janssen COVID-19 vaccine, and vice versa.⁶ If there is no other contraindication (for example; a proven allergy to polysorbate 80), the UK recommendation is that the AstraZeneca vaccine can be used as an alternative.[35]. 

### 7.2. Diagnostic procedures

Allergological study include detailed history and in vitro tests with the vaccines and their components. The sensitivity and specificity of skin tests with vaccines in confirming or discarding allergy to a vaccine or its ingredients have not been established.[8] But, if skin testing results negative, it is very unlikely for the patient to have IgE against the vaccine or its ingredients. If the suspected vaccine contains specific components known to be potentially allergenic, testing should also be conducted for those components. A prick test can be performed with the culprit vaccine solution as well as with PEG and polysorbate (titrated 10% and 1%). Currently the limited covid 19 vaccine supply, lack of information about sensitivity or specificity, and uncertain safety of the skin testing can interfere with skin testing with the vaccine. 

PEG and polysorbate skin testing may be of value in decision making around future COVID-19 vaccination. PEG 3350 is available as a laxative (miralax and others) for prick skin testing, and Refresh Optive Advanced Lubricant eye drops and Prevnar are an alternate source for polysorbate 80 skin testing.[36] Concentrations as high as 100% (17 g/17 mL) have stated to be nonirritating for prick skin testing.[40, 42] If the PEG prick skin test result is negative, an intradermal test can be performed; at first with a 1:100 dilution and, if negative, at a 1:10 dilution.

It is still unknown whether a negative prick test has sufficient positive or negative predictive power to determine the risk of a serious systemic reaction. Systemic reactions, including anaphylaxis, have been associated with both skin prick testing and intradermal testing.[36, 38] So these tests should be performed with evaluating the benefits and risks by experts in an appropriate settings. (Table 3)

**Table 3:** Recommendations for covid 19 vaccines.

Recommendations for covid 19 vaccines
Anaphylaxis is a severe, life-threatening allergic reaction that occurs rarely following vaccination.
Locations administering Covid-19 vaccines should have necessary supplies and staff members available to manage anaphylaxis and immediately treat suspected anaphylaxis with intramuscular adrenaline injection.
A history of food, venom, inhalant, latex, pet allergies, and allergy to oral medications (including the oral equivalent of an injectable medication) are not contraindications for receiving the covid 19 vaccines.
Mastocytosis/mast cell activation syndromes, idiopathic anaphylaxis, severe or multiple drug allergy and previous anaphylaxis to other vaccines are not considered contraindications for vaccination. These patients are recommended to be observed for 30 min after vaccination in a hospital setting.
Patients with only a delayed onset local reaction around the injection site area after the first vaccine dose do not have a contraindication for the future dose
In case of postvaccination allergic reaction, the patient should be referred to an allergy immunologist.
An allergic reaction occurs not only against the active ingredient itself but also against the vaccine components.
Skin prick tests should be performed by allergy-immunologists evaluating the benefits and risks in an appropriate settings.

## 8. Conclusion

Covid 19 virus has killed millions worldwide; however, only a very small number of patients have suffered from reactions after vaccination. It is very important that patients understand the overall benefit of vaccination against SARS-CoV-2, and they should be aware that an extremely low overall risk of allergic reactions is related. As allergists, we must be clear and evidence-based about allergic reaction risks with these vaccines. In near future, molecular diagnostic methods may also be used, and desensitisation protocols with vaccine or vaccine components can be applicable. The most important thing to keep in mind is that the benefits of the vaccine go far beyond the side effects, and most vaccine-related side effects are mild allergic reactions.

## Statement of ethics

This study is a review article. No human or animal experimental studies have been conducted. Therefore, no application was made to the ethics committee.

## References

[ref1] Delany I Rappuoli R 2014 Vaccines for the 21st century EMBO Molecular Medicine 6 708 720 24803000 10.1002/emmm.201403876 PMC4203350

[ref2] Whitney CG Zhou F Singleton J Schuchat A 1994 Benefits from immunization during the vaccines for children program era—United States, MMWR Morbidity and Mortality Weekly Report 63 2013 2013 PMC4584777 24759657

[ref3] McNeil MM Weintraub ES Duffy J Sukumaran L Jacobsen SJ 2016 Risk of anaphylaxis after vaccination in children and adults Journal of Allergy and Clinical Immunology 137 868 878 10.1016/j.jaci.2015.07.048 PMC4783279 26452420

[ref4] Kelso JM 2021 mRNA SARS-CoV-2/COVID-19 vaccines. Vaccine 39 865 865 33441235 10.1016/j.vaccine.2020.12.084 PMC7837118

[ref5] Murphy KR Patel NC Ein D Hudelson M Kodoth S Immunology Vaccine Task 2021 Force: Allergic Reactions to mRNA SARS-CoV-2 Vaccines: Allergic Reactions to mRNA SARS-CoV-2 Vaccines Asthma & Immunology 10 10.1016/j.anai.2021.01.017 PMC7825848 33493641

[ref6] COVID C Team R vaccine—United States 2020 Allergic reactions including anaphylaxis after receipt of the first dose of Pfizer-BioNTech COVID- December 14–23 70 46 46

[ref7] COVID C Team R. Allergic Vaccine—United States 2021 Reactions Including Anaphylaxis After Receipt of the First Dose of Moderna COVID- December 21 2020 2020

[ref8] Echeverría-Zudaire LA Ortigosa-del Castillo L Alonso-Lebrero E Álvarez-García FJ Cortés-Álvarez N 2015 Consensus document on the approach to children with allergic reactions after vaccination or allergy to vaccine components Allergologia et Immunopathologia 43 304 325 25891956 10.1016/j.aller.2015.01.004

[ref9] Simons FER Ebisawa M Sanchez-Borges M Thong BY Worm M 2015 update of the evidence base: World Allergy Organization anaphylaxis guidelines World Allergy Organization Journal 8 32 32 10.1186/s40413-015-0080-1 PMC4625730 26525001

[ref10] McNeil MM 2018 DeStefano F Journal of Allergy and Clinical Immunology 141 463 472 10.1016/j.jaci.2017.12.971 PMC6602527 29413255

[ref11] Stone Jr CA Rukasin CR Beachkofsky TM Phillips EJ 2019 Immune-mediated adverse reactions to vaccines British Journal of Clinical Pharmacology 85 2694 2706 31472022 10.1111/bcp.14112 PMC6955412

[ref12] da Silva EZM Jamur MC Oliver C 2014 Mast cell function: a new vision of an old cell Journal of Histochemistry & Cytochemistry 62 698 738 25062998 10.1369/0022155414545334 PMC4230976

[ref13] Porebski G Kwiecien K Pawica M Kwitniewski M 2018 Mas-related G protein-coupled receptor-X2 (MRGPRX2) in drug hypersensitivity reactions Frontiers in Immunology 9 3027 3027 30619367 10.3389/fimmu.2018.03027 PMC6306423

[ref14] Spoerl D Nigolian H Czarnetzki C Harr T. 2017 Reclassifying anaphylaxis to neuromuscular blocking agents based on the presumed patho-mechanism: IgE-mediated, pharmacological adverse reaction or “innate hypersensitivity International Journal of Molecular Sciences 18 1223 1223 10.3390/ijms18061223 PMC5486046 28590439

[ref15] Wood RA Berger M Dreskin SC Setse R Engler RJ 2008 An algorithm for treatment of patients with hypersensitivity reactions after vaccines Pediatrics 122 e771 e777 18762513 10.1542/peds.2008-1002

[ref16] Johansson S Hourihane JB Bousquet J Bruijnzeel-Koomen C Dreborg S 2001 A revised nomenclature for allergy: an EAACI position statement from the EAACI nomenclature task force Allergy 56 813 824 11551246 10.1034/j.1398-9995.2001.t01-1-00001.x

[ref17] Kelso JM Greenhawt MJ Li JT Nicklas RA Bernstein DI 2012 Adverse reactions to vaccines practice parameter 2012 update Journal of Allergy and Clinical Immunology 130 25 43 10.1016/j.jaci.2012.04.003 22608573

[ref18] Dreskin SC Halsey NA Kelso JM Wood RA Hummell DS 2016 International Consensus (ICON): allergic reactions to vaccines World Allergy Organization Journal 9 32 32 10.1186/s40413-016-0120-5 PMC5026780 27679682

[ref19] Muraro A Roberts G Worm M Bilò M Brockow K 2014 Anaphylaxis: guidelines from the E uropean A cademy of A llergy and C linical I mmunology Allergy 69 1026 1045 24909803 10.1111/all.12437

[ref20] Johansson S Bieber T Dahl R Friedmann PS Lanier BQ October 2003 Revised nomenclature for allergy for global use: Report of the Nomenclature Review Committee of the World Allergy Organization Journal of Allergy and Clinical Immunology 113 832 836 10.1016/j.jaci.2003.12.591 15131563

[ref21] 2018 Anaphylaxis; Turkish National Guideline 16

[ref22] Sampson HA Muñoz-Furlong A Campbell RL Adkinson Jr NF Bock SA 2006 Second symposium on the definition and management of anaphylaxis: summary report—Second National Institute of Allergy and Infectious Disease/Food Allergy and Anaphylaxis Network symposium Journal of Allergy and Clinical Immunology 117 391 397 10.1016/j.jaci.2005.12.1303 16461139

[ref23] Bohlke K Davis RL DeStefano F Marcy SM Braun MM 2004 Epidemiology of anaphylaxis among children and adolescents enrolled in a health maintenance organization Journal of Allergy and Clinical Immunology 113 536 542 10.1016/j.jaci.2003.11.033 15007358

[ref24] Brown SG. 2004 Clinical features and severity grading of anaphylaxis Journal of Allergy and Clinical Immunology 114 371 376 10.1016/j.jaci.2004.04.029 15316518

[ref25] Vanlander A Hoppenbrouwers K. 2014 Anaphylaxis after vaccination of children: review of literature and recommendations for vaccination in child and school health services in Belgium 32 3147 3154 10.1016/j.vaccine.2014.03.096 24726249

[ref26] Control CfD January 2005 MMWR Morbidity and Mortality Weekly Report 57 457 460 18451756

[ref27] Brown SG 2005 Cardiovascular aspects of anaphylaxis: implications for treatment and diagnosis Current Opinion in Allergy and Clinical Immunology 5 359 364 15985820 10.1097/01.all.0000174158.78626.35

[ref28] Caubet Clinics Allergy 2014 Vaccine Allergy 34 597 613 10.1016/j.iac.2014.04.004 25017679

[ref29] Bergfors E Trollfors B 2013 Sixty-four children with persistent itching nodules and contact allergy to aluminium after vaccination with aluminium-adsorbed vaccines—prognosis and outcome after booster vaccination European Journal of Pediatrics 172 171 177 23052615 10.1007/s00431-012-1841-2

[ref30] Nilsson L Brockow K Alm J Cardona V Caubet JC 2017 Vaccination and allergy: EAACI position paper, practical aspects Pediatric Allergy and Immunology 28 628 640 28779496 10.1111/pai.12762

[ref31] Jackson LA Starkovich P Dunstan M Yu O Nelson J 2008 Prospective assessment of the effect of needle length and injection site on the risk of local reactions to the fifth diphtheria-tetanus-acellular pertussis vaccination Pediatrics 121 e646 e652 18310184 10.1542/peds.2007-1653

[ref32] Jackson LA Peterson D Nelson JC Marcy SM Naleway AL 2013 Vaccination site and risk of local reactions in children 1 through 6 years of age Pediatrics 131 283 289 23319538 10.1542/peds.2012-2617

[ref33] 2011 General recommendations on immunization---recommendations of the Advisory Committee on Immunization Practices (ACIP) MMWR Recommendations and reports: Morbidity and mortality weekly report Recommendations and reports 60 1 64 21293327

[ref34] Dreskin SC Halsey NA Kelso JM Wood RA Hummell DS 2016 International Consensus (ICON): allergic reactions to vaccines World Allergy Organization Journal 9 1 21 10.1186/s40413-016-0120-5 PMC5026780 27679682

[ref35] Turner PJ Ansotegui IJ Campbell DE Cardona V Ebisawa M 2021 -19 Vaccine-associated anaphylaxis: a statement of the world allergy organization anaphylaxis committee World Allergy Organization Journal 100517 10.1016/j.waojou.2021.100517 PMC7857113 33558825

[ref36] Banerji A Wickner PG Saff R Robinson LB 2020 Disease and Reported Allergic Reactions: Current Evidence and Approach 10 10.1016/j.jaip.2020.12.047 PMC7948517 33388478

[ref37] Kleine-Tebbe J Klimek L Hamelmann E Pfaar O Taube C 2021 Severe allergic reactions to the COVID-19 vaccine–statement and practical consequences Allergologie Select 5 26 26 33426427 10.5414/ALX02215E PMC7787363

[ref38] Sellaturay P Nasser S Ewan P. Polyethylene Glycol–Induced Systemic Allergic Reactions 2021 The Journal of Allergy and Clinical Immunology: In Practice 9 670 675 33011299 10.1016/j.jaip.2020.09.029

[ref39] Bruusgaard-Mouritsen MA Johansen JD Garvey LH. Clinical manifestations and impact on daily life of allergy to polyethylene glycol (PEG) in ten patients Clinical & Experimental Allergy. doi: 10 10.1111/cea.13822 33394522

[ref40] Wenande E Garvey L 2016 Immediate-type hypersensitivity to polyethylene glycols: a review Clinical & Experimental Allergy 46 907 922 27196817 10.1111/cea.12760

[ref41] Zhou Z-H Jakubovic B Phillips EJ Sussman G 2020 Anti-PEG IgE in anaphylaxis associated with polyethylene glycol The Journal of Allergy and Clinical Immunology: In Practice 10 10.1016/j.jaip.2020.11.011 PMC8090930 33217616

[ref42] Liu Y Relling MV Krantz MS Pratt AL 2019 Immediate hypersensitivity to polyethylene glycols and polysorbates: more common than we have recognized The Journal of Allergy and Clinical Immunology: In Practice 7 1533 1540 30557713 10.1016/j.jaip.2018.12.003 PMC6706272

[ref43] Fruijtier-Pölloth C 2005 Safety assessment on polyethylene glycols (PEGs) and their derivatives as used in cosmetic products Toxicology 214 1 38 16011869 10.1016/j.tox.2005.06.001

[ref44] Sahiner UM Yavuz ST Gökce M Buyuktiryaki B Altan I 2013 Anaphylactic reaction to polyethylene-glycol conjugated-asparaginase: Premedication and desensitization may not be sufficient Pediatrics International 55 531 533 23910807 10.1111/ped.12131

[ref45] Szebeni J Fontana JL Wassef NM Mongan PD Morse DS 1999 Hemodynamic changes induced by liposomes and liposome-encapsulated hemoglobin in pigs: a model for pseudoallergic cardiopulmonary reactions to liposomes: role of complement and inhibition by soluble CR1 and anti-C5a antibody Circulation 99 2302 2309 10226097 10.1161/01.cir.99.17.2302

[ref46] Mohamed M Abu Lila AS Shimizu T Alaaeldin E Hussein A 2019 PEGylated liposomes: immunological responses Science and Technology of Advanced Materials 20 710 724 31275462 10.1080/14686996.2019.1627174 PMC6598536

[ref47] Coors EA Seybold H Merk HF Mahler V 2005 Polysorbate 80 in medical products and nonimmunologic anaphylactoid reactions Annals of Allergy, Asthma & Immunology 95 593 599 10.1016/S1081-1206(10)61024-1 16400901

[ref48] Hawe A Filipe V Jiskoot W. Fluorescent 2010 molecular rotors as dyes to characterize polysorbate-containing IgG formulations Pharmaceutical Research 27 314 326 20041280 10.1007/s11095-009-0020-2 PMC2812426

[ref49] Cabanillas B Akdis C Novak N. 2020 Allergic reactions to the first COVID-19 vaccine: a potential role of Polyethylene glycol 10 10.1111/all.14711 33320974

[ref50] Sokolowska M Eiwegger T Ollert M Torres MJ Barber D EAACI statement on the diagnosis, management and prevention of severe allergic reactions to COVID-19 vaccines 10 10.1111/all.14739 PMC8013422 33452689

[ref51] Tanno LK Gonzalez-Estrada A Olivieri B Caminati M. Asthma 2019 and anaphylaxis Current Opinion in Allergy and Clinical Immunology 19 447 455 31261185 10.1097/ACI.0000000000000566

[ref52] Shaker MS Wallace DV Golden DB Oppenheimer J Bernstein JA 2020 Anaphylaxis—a 2020 practice parameter update, systematic review, and Grading of Recommendations, Assessment, Development and Evaluation (GRADE) analysis Journal of Allergy and Clinical Immunology 145 1082 1123 10.1016/j.jaci.2020.01.017 32001253

[ref53] Perel P Roberts I. 2012 Colloids versus crystalloids for fluid resuscitation in critically ill patients Cochrane Database of Systematic Reviews 6 10.1002/14651858.CD000567.pub5 22696320

[ref54] Cardona V Ansotegui IJ Ebisawa M El-Gamal Y Rivas MF 2020 World allergy organization anaphylaxis guidance 2020. World Allergy Organization Journal 13 100472 100472 10.1016/j.waojou.2020.100472 PMC7607509 33204386

[ref55] Pumphrey R 2000 Lessons for management of anaphylaxis from a study of fatal reactions Clinical and Experimental Allergy 30 1144 1150 10931122 10.1046/j.1365-2222.2000.00864.x

[ref56] Simons FER Ardusso LR Bilò MB El-Gamal YM Ledford DK 2011 World allergy organization guidelines for the assessment and management of anaphylaxis World Allergy Organization Journal 4 13 37 10.1097/WOX.0b013e318211496c PMC3500036 23268454

[ref57] Michelson KA Monuteaux MC Neuman MI 2015 Glucocorticoids and hospital length of stay for children with anaphylaxis: a retrospective study The Journal of Pediatrics 167 719 724 26095285 10.1016/j.jpeds.2015.05.033

[ref58] Alqurashi W Ellis AK 2017 Do corticosteroids prevent biphasic anaphylaxis? The Journal of Allergy and Clinical Immunology: In Practice 5 1194 1205 28888249 10.1016/j.jaip.2017.05.022

[ref59] Thomas M Crawford I. 2005 Glucagon infusion in refractory anaphylactic shock in patients on beta-blockers Emergency Medicine Journal 22 272 273 15788828 10.1136/emj.2005.023507 PMC1726748

